# The Value of Vaccination

**Published:** 1920-04-17

**Authors:** 


### THE VALUE OF VACCINATION.

Our attention is drawn to a somewhat bitter and, in parts, confusing correspondence which has been carried on in the pages of the *Hereford Times*, and taking it as a typical instance of the methods by which the public, particularly the provincial public, are being urged to refuse vaccination for their children and themselves, we feel that a few comments on the situation and facts as they really are will not at the moment be out of place.

It is difficult to choose the most appropriate anti-vaccination fallacy which merits attack, but the most surprising, the most startling, in fact, to the average medical critic, is that apparently doubt is cast upon the production of any artificial immunity at all. In face of the findings of the Royal Commission on vaccination in 1896, this attempted confusion of the rational measures which should be urged by health authorities is not only futile, but absolutely unjustifiable. A commission fully qualified to judge decided at that time, among other points, that vaccination modifies the character of smallpox and renders it less fatal and of a less severe type. Also that the person's liability to be attacked is lessened. Further, that this power to modify the character of the disease is greatest in the period in which its power to protect from attack is greatest, but that its power thus to modify does not diminish as rapidly as its protective influence, the efficiency towards changing the result of infection into a mild type of disease remaining in the later periods of life. And, finally, two most important points were made. That re-vaccination restores the protection which lapse of time has diminished, and that the beneficial effects of vaccination are most experienced by those in whose case it has been most thorough.

Time and later experience have failed to disprove any of these findings.

More recently the late Sir William Osler summed up the situation not only of old, densely populated England, but of America, with her increased opportunities of sanitary living, when he said, "In an immense majority of these cases successful inoculation renders the person for many years unsusceptible. Communities in which vaccination and re-vaccination are successfully carried out are those in which smallpox has the fewest victims."

If we are going to ply the public with medical facts, let us cite the major statistics, the big issues in the history of vaccination. Let us remind them of Germany, surrounded on three sides by badly-vaccinated countries, who succeeded, until the war upset much of the smooth working of her health control, in almost stamping out smallpox as a serious disease. In Germany vaccination and re-vaccination were compulsory, and as a result, in Berlin, with a population of 2,000,000, twelve hospital beds reserved for smallpox cases have been found for many years to meet all requirements! Of the few cases which did occur, over 70 per cent. were resident on the frontiers, while in Austria and Belgium the smallpox death-rates were over twenty times as great as in Germany.

Isolated references to 1857, 1871, and other dates so far back are not only misleading but absolutely useless when comparative deductions made from happenings at that time are directly applied to the conditions of 1920. Whatever the imperfections of to-day, disease-control measures in Britain are at least an immense advance on those of half a century ago.

Contradictory interpretations can be obviously but very wrongly drawn from the statistics and circumstances of those times, and let us appreciate that our present health figures are built up on the gradual elimination of disease which occurred as control improved, and in no instance is this better exemplified than in the progressive reduction, first, of the prevalence and, later, of the virulence of smallpox infections. Recently the whole matter has been ably set out by Dr. J. C. McVail, and if any wish to appreciate the true depth of absurdity in the fallacies of such anti-vaccination arguments as we mentioned at the beginning of this note, we would refer them to Dr. McVail's writings, which are now published in book form. To us it seems undeniable that for the relative freedom of this country from smallpox and, therefore, for the very fact that they can for one moment justify their more extreme exhortations, the anti-vaccination enthusiasts have to thank the very process of immunisation which they actively but most inadvisedly condemn.

